# Supplementary material for: Methods for the targeted sequencing and analysis of integrons and their gene cassettes from complex microbial communities
Source: Microb Genom. 2022 Mar 17;8(3):000788. doi: 10.1099/mgen.0.000788 (PMC9176274; doi:10.1099/mgen.0.000788)
Supplement: Supplementary material 1 [file mgen-8-0788-s001.pdf]

Supplementary Figures

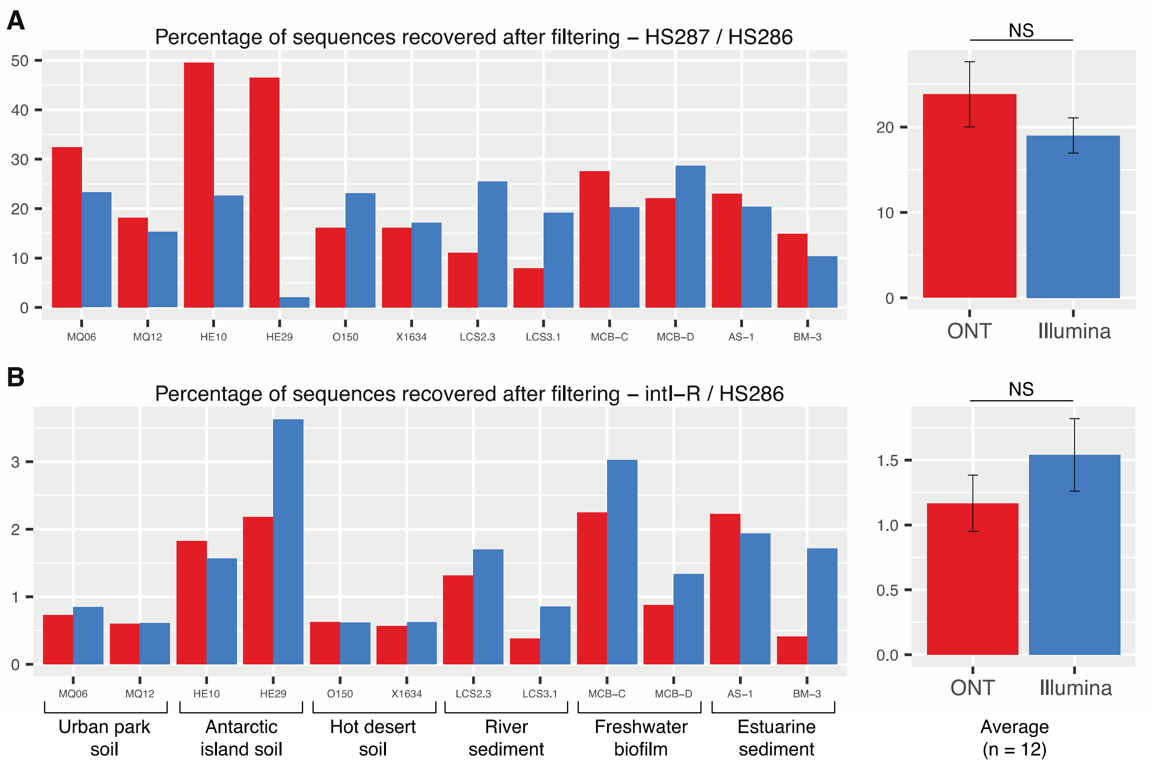

Figure S1. Percentages of sequences recovered after bioinformatic filtering. Filtering involved screening (**A**) HS287 / HS286 sequences for cassette recombination sites (*attC*s) and (**B**) intI-R / HS286 sequences for integron integrase (IntI) encoding genes to ensure that they represented amplicons of genuine integrons. Average (± 1 S.E) diversity for each analysis are shown on the right-hand side of each panel. Differences between Nanopore (ONT) and Illumina MiSeq technologies were not significant (NS) as determined by two-sample T-tests.

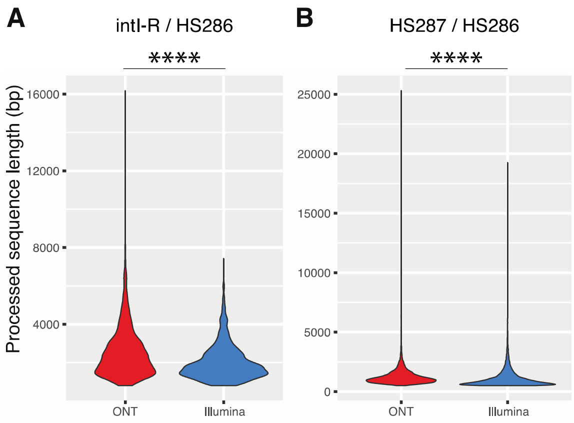

Figure S2. Processed sequence lengths recovered by each sequencing technology. The violin plots show the range of sequence lengths (bp) for (**A**) intI-R / HS286 and (**B**) HS287 / HS286 primer sets. The width of each curve represents the relative density of datum points across the ranges. For both primer sets, the average length of recovered amplicons (n=12) is significantly larger (Wilcoxon rank sum test, P<0.0001) when sequenced with Nanopore (ONT) compared to Illumina sequencing.

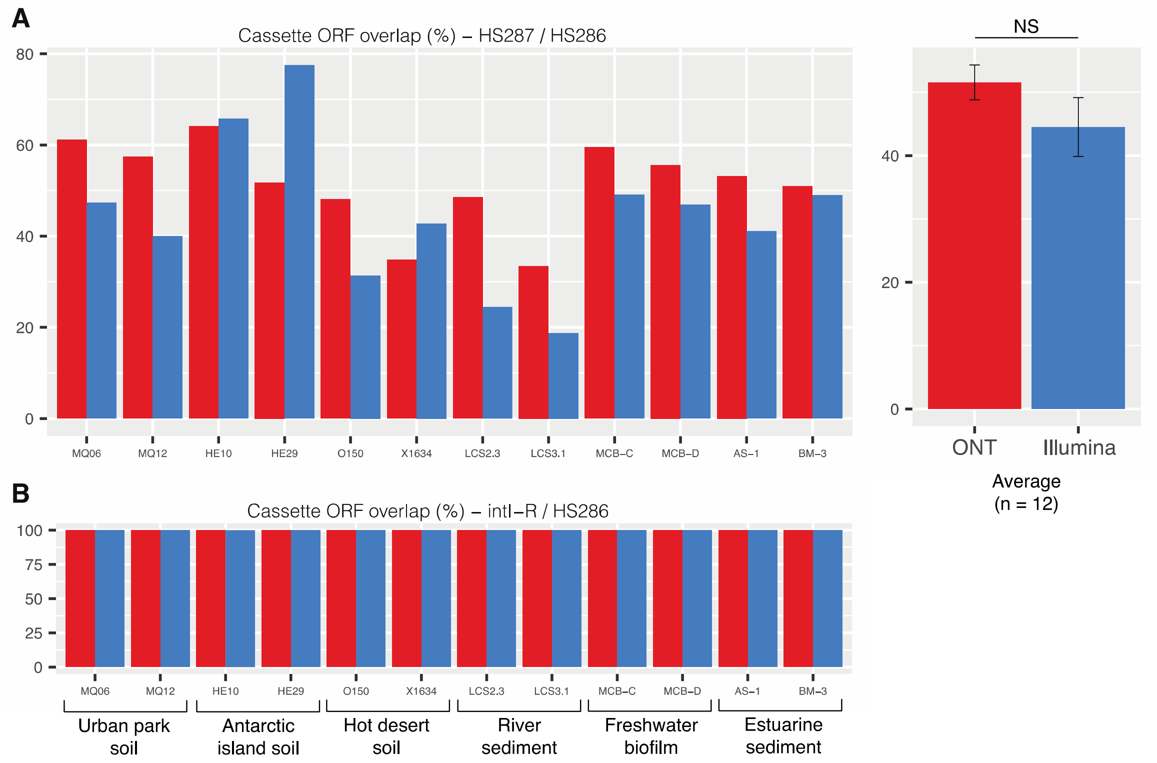

Figure S3. Overlap in recovered ORFs between Nanopore (ONT) and Illumina sequencing technologies. The percentage of ORFs recovered from one sequencing technology that were covered by reads from the other technology are shown for (**A**) HS287 / HS286 and (**B**) intI-R / HS286 primer sets. ORFs considered to present in the opposite sequencing technology had to have a mean coverage depth of at least 1x that spanned at least 98% of the ORF. The average (± 1 S.E) percentage overlap for HS287 / HS286 data is shown on the right-hand side of panel (**A**). There was no significant (NS) difference between ONT and Illumina (Two-sample T-test, P=0.209).

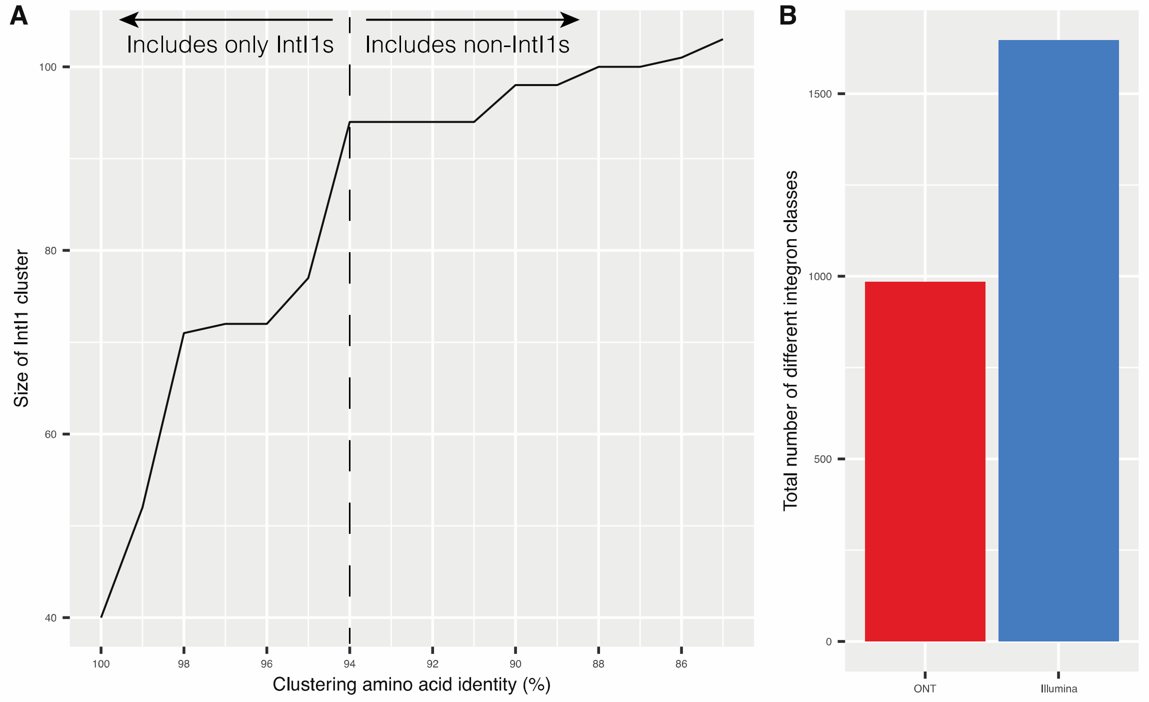

Figure S4. Diversity of integron classes. (**A**) The amino acid clustering threshold for integron classes was determined using class 1 integron integrases (IntI1) present in our dataset. Decreasing amino acid clustering thresholds were iteratively set until all IntI1s were grouped in the same cluster and all non-IntI1s were excluded. A protein sequence was considered to be IntI1 if it aligned with any previously characterised class 1 integron in GenBank using BLASTP (>98% amino acid identity and >70% subject cover). An amino acid clustering threshold of 94% was found to include all IntI1s (n=94) and exclude all non-IntI1s. (**B**) The total number of integron classes (based on a 94% amino acid clustering threshold) recovered for all samples (n=12).

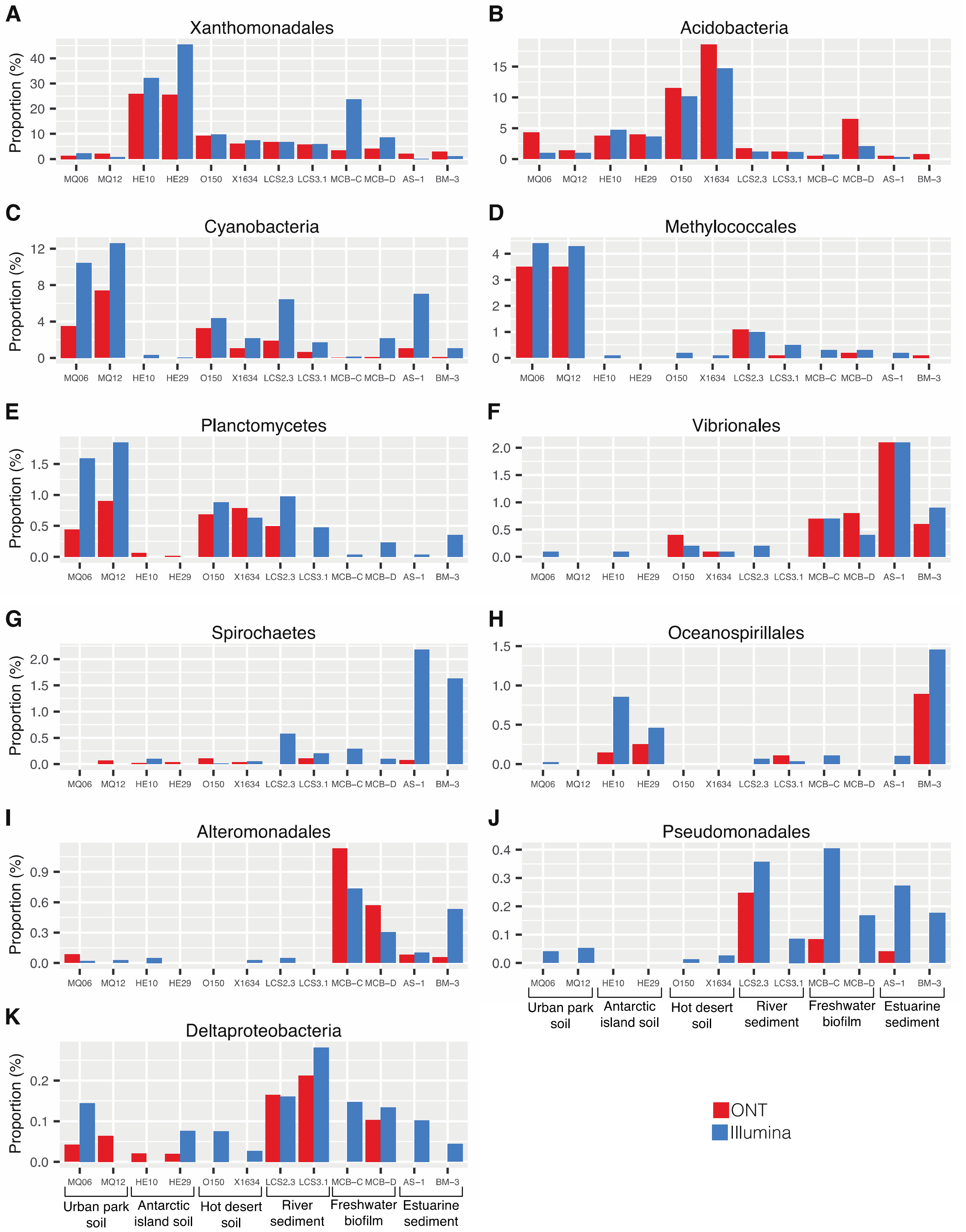

Figure S5. Proportions of gene cassette recombination sites (*attC*s) assigned to bacterial taxa. Taxonomic predictions are based on all eleven (**A-K**) available taxonomic models of chromosomal *attC*s. Each figure panel shows the proportion of *attC*s across each sample that exhibit sequence and structure conserved among that taxon.
